# *Capsosiphon fulvescens* glycoprotein reduces AGS gastric cancer cell migration by downregulating transforming growth factor-β _1_ and integrin expression

**DOI:** 10.3892/ijo.2013.2055

**Published:** 2013-08-07

**Authors:** YOUNG-MIN KIM, IN-HYE KIM, TAEK-JEONG NAM

**Affiliations:** Institute of Fisheries Sciences, Pukyong National University, Gijang-gun, Busan 619-911, Republic of Korea

**Keywords:** capsosiphon fulvescens, transforming growth factor-β _1
_, FAK/AKT/small GTPase, integrin, cell migration

## Abstract

Seaweeds are commonly used as functional foods and drugs. A glycoprotein (GP) from the green alga *Capsosiphon fulvescens* (Cf) has been reported to have anti-tumor activity toward various cancer cells. We previously observed that Cf-GP induced different pathways of apoptosis in AGS human gastric cancer cells. Transforming growth factor (TGF)-β _1_ plays an important role in cancer cell migration. Increased TGF-β _1_ levels increase the expression of the small GTPases and activate the FAK/PI3K/AKT pathways, resulting in the upregulation of integrin receptor proteins, which mediate the attachment of cells to surrounding tissues, cells or extracellular matrix. Thus, the inhibition of TGF-β _1_ signaling would downregulate integrin expression and thereby effectively decrease cell growth and migration. In the present study, we determined the effect of Cf-GP treatment on the proliferation, migration and apoptosis of AGS human gastric cancer cells. To investigate the mechanism by which Cf-GP exerts its anticancer actions, we examined the effect of Cf-GP on the expression levels of TGF-β _1_ , FAK, PI3K, AKT, the small GTPases and integrins in AGS cells. Our findings indicate that Cf-GP inhibits AGS cell proliferation and migration by downregulating integrin expression via the TGF-β _1_ -activated FAK/PI3K/AKT pathways. These results suggest that Cf-GP may be an important factor in the development of functional foods and therapeutic agents.

## Introduction

*Capsosiphon fulvescens* (Cf) is green seaweed that grows mainly in the clean areas of the sea off the coast of Korea. It is commonly used as a traditional health food, and various bioactive effects of Cf have been reported ( 1 ) . A compositional analysis of Cf showed that it contains high levels of proteins, carbohydrates and amino acids ( 2 ) . Specific substances in Cf are known to boost the immune system, bio-activity and have anticancer activities ( 3 – 5 ) . We previously observed the induction of apoptosis in AGS human gastric cancer cells by a glycoprotein of Cf ( 6 , 7 ) . This glycoprotein has also been reported to inhibit cell invasion ( 8 ) .

Transforming growth factor (TGF)-β _1_ is a cytokine associated with various human cancers ( 9 , 10 ) involving macrophages, brain cells and keratinocytes. Previous studies have reported that TGF-β _1_ modulates cell migration, invasion and proliferation in gastric cancer cells ( 11 ) . TGF-β _1_ induces the overexpression of growth factor focal adhesion kinase (FAK) protein in several cancers, activates phosphatidylinositol 3-kinase (PI3K)/AKT and small GTPase proteins ( 12 ) , and upregulates integrin proteins. The small GTPases, which include Rho A, Rho B, Rac-1 and Cdc 42, are involved in the signaling pathways associated with diverse cellular functions, including cell proliferation and migration, in response to different growth factor receptors ( 13 ) . The small GTPases also activate nuclear transcription factor-κB (NF-κB), which upregulates the expression of integrin receptor proteins, thereby contributing to cell migration.

Integrin receptors are located on the cell surface, where they are responsible for the adhesion of cells to extracellular matrix (ECM) proteins such as fibronectin and the transduction of extracellular signals to the cells ( 14 ) . Integrins exist as hetero dimers of two distinct transmembrane glycoprotein chains, called α and β subunits, that are non-covalently linked. The integrin family consists of 24 different αβ heterodimers ( 15 ) . The binding of an integrin receptor to its extracellular ligand causes a signal to be relayed into the cell, resulting in the regulation of specific gene expression ( 16 ) . The binding of integrin receptors to ECM molecules also produces cell adhesion, which is critical for cell migration, proliferation and differentiation.

The present study investigated the possible relationship between the anticancer activity of the *C. fulvescens* glycoprotein (Cf-GP) and the downregulation of integrin expression via the TGF-β _1_ -activated PI3K/AKT/small GTPases pathway in AGS human gastric cancer cells. First, we established the effect of Cf-GP treatment on the proliferation, migration and apoptosis of AGS cells. Then, to investigate the mechanism by which Cf-GP may exert its anticancer activity, we examined the effect of Cf-GP on the expression levels of TGF-β _1_ , the small GTPases, and integrins in the AGS cells.

## Materials and methods

### Preparation of Cf-GP

The *C. fulvescens* used in this experiment was purchased in 2010 in Republic of Korea. The Cf powder (40 g) was diluted with water (1 liter) and stirred for 3 h at 80°C in a heating mantle. The solution was clarified by centrifugation at 1,500 × g for 15 min at 4°C. Three volumes of 95% ethanol were added to the solution, and precipitates were removed by vacuum filtration. The supernatant was mixed with 80% ammonium sulfate and stirred for 24 h, followed by dialysis (Por Membrane MW 3,500 Da, Spcectrum Laboratories Inc., Rancho Dominguez, CA, USA) for one day at 4°C to remove salts. The concentrated solution was distributed into 1.5-ml tubes and stored at −70°C until use. These samples were named Cf-GP.

### Cell culture

AGS human gastric cancer cell line (American Type Culture Collection, Manassas, VA, USA) was cultured in RPMI-1640 medium with 10% fetal bovine serum (FBS; Hyclone, Logan, UT, USA), 100 U/ml penicillin, and 100 mg/ml streptomycin, at a temperature of 37°C in a humidified atmosphere of 5% CO _2_ . The cells were cultured to 80% confluence in 100-mm dishes. The medium was replaced daily.

### Cell proliferation assay

AGS cell proliferation was measured using a CellTiter 96 ^®^ aqueous non-radioactive cell proliferation assay (Promega, Madison, WI, USA), which is based on the cleavage of 3-(4,5-dimethylthiazol-2-yl)-5-(3-carboxymethoxyphenyl)-2-(4-sulfonyl)-2H-tetrazolium (MTS) into a formazan product soluble in tissue culture medium. The cells were seeded onto 96-well plates at 2×10 ^4^ cells/well and the medium was replaced with serum-free medium (SFM) after culture for 24 h. After another 24 h, the medium was replaced with SFM containing Cf-GP (5, 10 and 20 *μ* g/ml), followed by incubation for 24 h. For the assay, MTS solution was added to the cells in each well and allowed to react for 30 min at 37°C. The absorbance of the solution in each well was measured at 490 nm using a microplate reader (Benchmark microplate reader; Bio-Rad Laboratories, Hercules, CA, USA).

### Cell migration assay

AGS cells were seeded onto 100-mm dishes and grown to 80% confluence. The medium was replaced with SFM, and the cells were cultured for 24 h, after which the cells were wounded by scraping with a pipette tip. The medium was replaced with SFM containing Cf-GP (5, 10 and 20 *μ* g/ml), and the cells were cultured for 24 h. Wound closure was determined from photographs taken using a microscope at ×200 magnification.

### Apoptosis assay

The level of apoptosis induced by Cf-GP treatment was determined using a Muse™ Annexin V and Dead Cell kit (EMD Millipore Co., Hayward, CA, USA). Cells were cultured in 6-well seeds to 60% confluency, and then the medium was replaced with SFM or SFM containing Cf-GP (5, 10 and 20 *μ* g/ml). After 24 h, the cells were collected in 1% FBS-RPMI-1640 medium, mixed with the Muse Annexin V and Dead Cell Reagent, and analyzed using a Muse Cell Analyzer (EMD Millipore Co.).

### mRNA expression assay

The mRNA expression levels of specific genes were evaluated by reverse-transcription polymerase chain reaction (RT-PCR). AGS cells were seeded onto 6-well plates at 2×10 ^4^ cells/well and were cultured for 24 h, after which the medium was replaced with SFM containing Cf-GP (5, 10 and 20 *μ* g/ml) for 24 h. Total RNA was isolated from the cells using TRIzol reagent (Invitrogen Co., Carlsbad, CA, USA), and total RNA was converted to cDNA using oligo(dT) primers (iNtRON Biotechnology Inc., Seongnam, Korea). For PCR amplification, the cDNA and specific primers ( Table I ) were added to 2X TOPsimple™ DyeMIX-nTaq (Enzynomics, Inc., Daejoen, Korea) and 0.1% diethylpyrocarbonate (DEPC) water. The amplified products were analyzed on 1% agarose gels stained with RedSafe™ nucleic acid staining solution (iNtRON Biotechnology, Inc.).

### Western blot analysis

AGS cells in 100-mm dishes were cultured to 80% confluence and then incubated in SFM for 4 h. Fresh SFM containing Cf-GP (5, 10 and 20 *μ* g/ml) was added to the cells, and the incubation continued for 24 h, after which the cells were washed with phosphate-buffered saline (PBS) and mixed with lysis extraction buffer [20 mM Tris (pH 7.5), 150 mM NaCl, 1% Triton X-100, 1 mM EDTA, 1 mM EGTA, 2.5 mM sodium pyrophosphate, 1 mM β-glycerophosphate, 1 mM sodium orthovanadate, 1 *μ* g/ml aprotinin, 1 *μ* g/ml leupeptin, 1 *μ* g/ml pepstatin A, 0.25% Na-deoxycholate, and 1 mM PMSF]. For western blot analysis, the cell lysates were electrophoresed in 10–15% polyacrylamide gels, and the resolved proteins were transferred to Immobilon-P transfer membrane (Millipore Co., Billerica, MA, USA). The membranes were blocked with 1% bovine serum albumin in TBS-T [10 mM Tris-HCl (pH 7.5), 150 mM NaCl, and 0.1% Tween-20] at room temperature, followed by incubation with the following specific primary antibodies (diluted 1:1,000): anti-TGF-β _1_ , anti-FAK, anti-PI3K, anti-AKT, anti-Rho A, anti-Rho B, anti-Rac-1, anti-Cdc 42, anti-NF-κB, anti-IκB, anti-integrin α _ν_ , anti-integrin β _1_ , anti-integrin β _3_ , and anti-integrin β _5_ . All primary antibodies were purchased from Santa Cruz Biotechnology Inc. (Santa Cruz, CA, USA). The secondary antibody was horse-radish peroxidase conjugated goat, mouse or rabbit antibody (1:10,000) (GE Healthcare Bio-Sciences, Piscataway, NJ, USA). Immunoreactive bands were detected with SuperSignal West Pico Chemiluminescent substrate (Thermo Fisher Scientific Inc., Rockford, IL, USA) and visualized on Kodak X-ray film.

### Statistical analysis

Data were analyzed using ANOVA. Values of p<0.05 on Duncan’s multiple range test indicated a significant difference between each groups. The results are presented as means ± SD. All analyses were performed with SPSS software (ver. 10.0; SPSS Inc., Chicago, IL, USA).

## Results

### Cf-GP inhibits AGS cell proliferation

**Figure 1.**
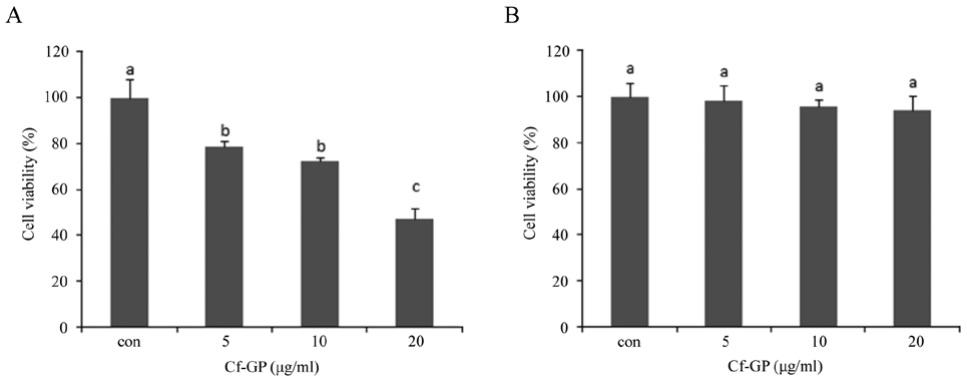
Cf-GP inhibits the proliferation of AGS cells. Cells were treated with Cf-GP (5, 10 and 20 *μ* g/ml) for 24 h. As a control, (A) AGS cell and (B) IEC-6 cell were similarly treated with Cf-GP. Cell proliferation was determined using a MTS assay. Values represent means ± SD. p<0.05 by ANOVA. Values with different letters are significantly different according to Duncan’s multiple range test.

The effects of Cf-GP on AGS cell proliferation were examined using the MTS assay. AGS cells treated with Cf-GP at 5, 10 and 20 *μ* g/ml showed dose-dependent inhibition of cell proliferation, with a maximum inhibition of 50% at 20 *μ* g/ml ( Fig. 1A ). We found Cf-GP dose-dependent effect of AGS cell growth inhibition by MTS assay. In addition we determined the toxicity of the normal cells. Treatment with Cf-GP had no toxic effects on the normal human intestinal epithelial IEC-6 cells ( Fig. 1B ).

### Cf-GP inhibits AGS cell migration

**Figure 2.**
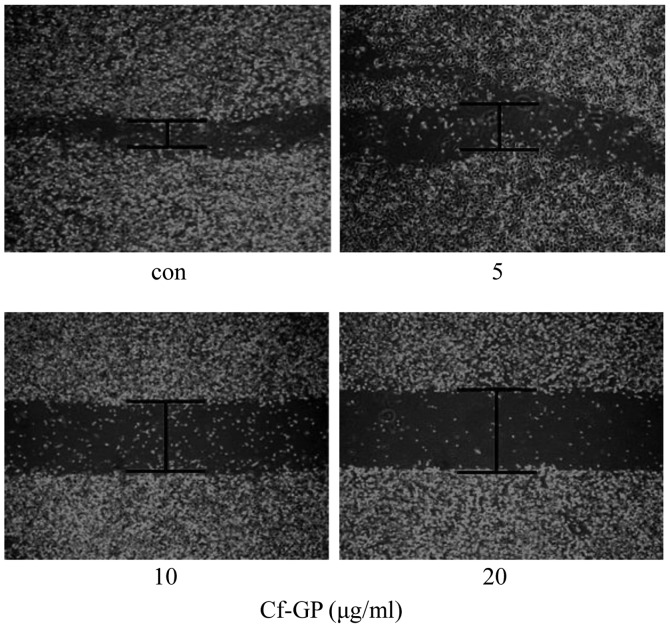
Cf-GP inhibits wound-healing migration of AGS cells. Cells were cultured in 100-mm dishes for 24 h, and then the cell layer was wounded by scraping. The medium was replaced with medium containing 1% FBS and Cf-GP (5, 10 and 20 *μ* g/ml). The denuded zone of cells was photographed using a microscope at ×200 magnification, and the degree of recovery was measured.

The effect of Cf-GP on AGS cell migration was examined using a wound-healing assay. As results, in the case of control AGS cells group over time, the mobility of cells in the wounded area was found to increase. But, AGS cells treated with Cf-GP (5, 10 and 20 *μ* g/ml) exhibited dose-dependent inhibition of migration into the cell-wounded zone on 100-mm dishes, indicating a Cf-GP-induced inhibition of cell mobility ( Fig. 2 ).

### Cf-GP dose-dependently increases cellular apoptosis in AGS cells

**Figure 3.**
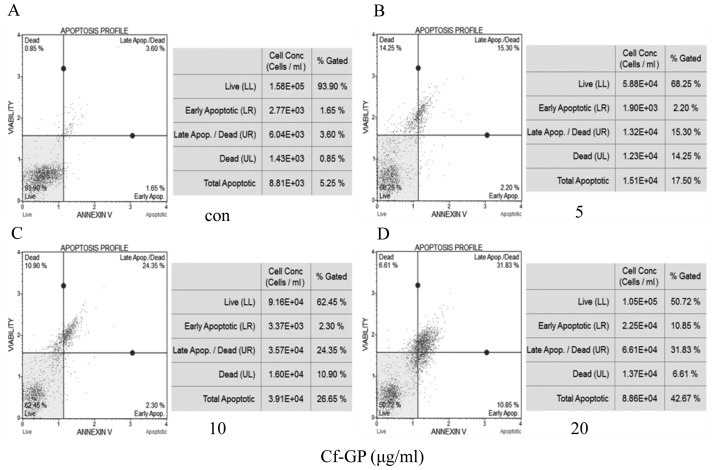
Cf-GP induces apoptosis of the AGS cells. Cells cultured in 6-well plates were treated with Cf-GP (5, 10 and 20 *μ* g/ml) and then collected in medium containing 1% FBS. The percentages of apoptotic and necrotic cells were determined using a Muse Annexin V and Dead Cell kit as described in the Materials and methods. Cells in the early stage of apoptosis are Annexin V-positive and 7-AAD-negative, and those in late apoptosis are Annexin V-positive and 7-AAD-positive.

AGS cells were treated with Cf-GP (5, 10 and 20 *μ* g/ml), and apoptotic and necrotic cells were detected by Annexin V and 7-aminoactinomycin D (AAD) staining, respectively ( 17 ) . In this assay, cells in the early stage of apoptosis are Annexin V-positive and 7-AAD-negative, and those in late apoptosis are Annexin V-positive and 7-AAD-positive. In the present study, Cf-GP treatment increased the percentage of apoptotic AGS cells, in a dose-dependent manner. Control cells comprised 5.25% apoptotic cells, 0.85% necrotic cells and 93.90% living cells ( Fig. 3 ). Treatment with 5, 10 and 20 *μ* g/ml Cf-GP for 24 h resulted in 17.50, 26.65 and 42.68% apoptotic cells, respectively.

### Cf-GP dose-dependently alters the expression of TGF-β _1_ and small GTPases

**Figure 4.**
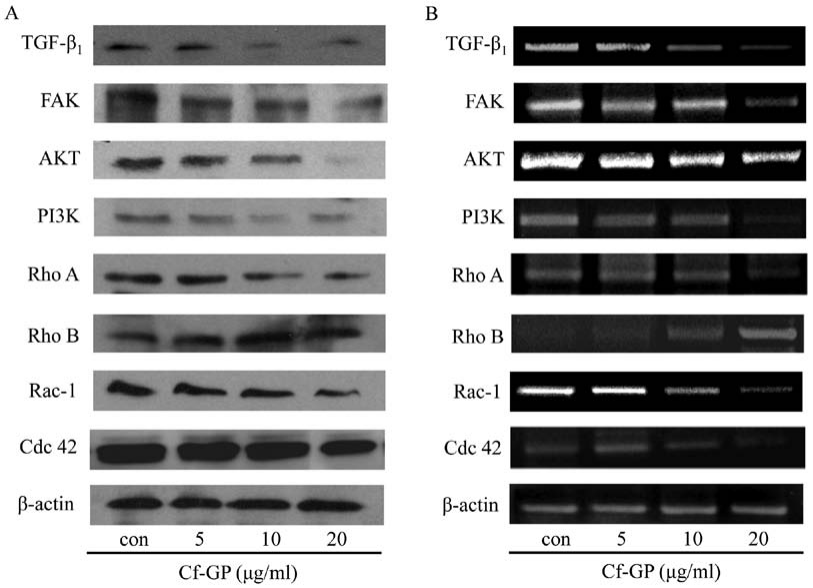
Cf-GP dose-dependently alters the expression of growth-regulating factors in AGS cells. (A) Protein and (B) mRNA expression levels of TGF-β _1_ , FAK, PI3K, AKT and the small GTPases Rho A, Rho B, Rac-1 and Cdc 42 in Cf-GP-treated (5, 10 and 20 *μ* g/ml). AGS cells were determined by western blot analysis and RT-PCR analysis, respectively, as described in Materials and methods.

The TGF-β _1_ receptor activates small GTPases and the FAK/PI3K/AKT pathways. The overexpression of FAK/PI3K/AKT can be induced by several other growth factors as well, and the Rho family of small GTPases plays a critical role in cancer cell growth and migration ( 18 , 19 ) . Thus, the altered expression of proteins associated with growth factors regulates cancer cell growth and migration ( 20 ) . In the present study, the protein and mRNA expression levels of TGF-β _1_ , FAK, PI3K, AKT and the small GTPases Rho A, Rho B, Rac-1 and Cdc 42 were determined by western blot analysis and RT-PCR analysis of AGS cells treated with Cf-GP (5, 10 and 20 *μ* g/ml) for 24 h. Treatment with Cf-GP dose-dependently downregulated the protein ( Fig. 4A ) and mRNA expression levels ( Fig. 4B ) of TGF-β _1_ , FAK, PI3K, AKT and the small GTPases Rho A, Rac-1 and Cdc 42. In contrast, Cf-GP treatment upregulated the protein and mRNA expression levels of Rho B, a small GTPase that is involved in the inhibition of cancer cell growth.

### Cf-GP dose-dependently downregulates the expression of NF-κB and IκB

**Figure 5.**
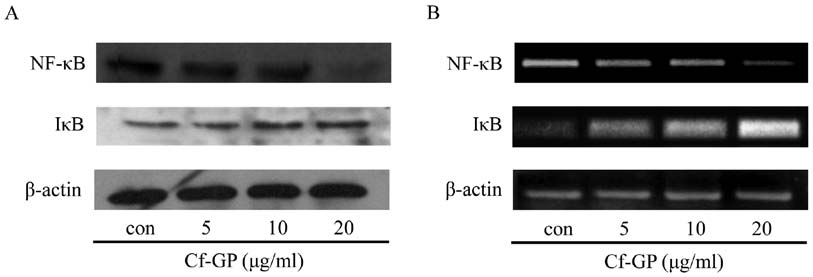
Cf-GP dose-dependently downregulates the expression of NF-κB and downregulates of IκB. (A) Protein and (B) mRNA expression levels of NF-κB and IκB in Cf-GP-treated (5, 10 and 20 *μ* g/ml) AGS cells were determined by western blot analysis and RT-PCR analysis, respectively, as described in the Materials and methods.

NF-κB activation is necessary for cancer cell growth and migration ( 21 ) . NF-κB inhibits activity through forming an NF-κB/IκB complex by upregulation of IκB, however, almost all cancer cells show downregulation IκB. Thus, degradation of NF-κB/IκB induced nuclear translocation of NF-κB movement. The protein and mRNA expression levels of the transcription factor NF-κB and its regulatory protein IκB in AGS cells treated with Cf-GP (5, 10 and 20 *μ* g/ml) for 24 h were analyzed by western blot analysis and RT-PCR. Cf-GP treatment downregulated the expression of NF-κB and upregulation of IκB ( Fig. 5 ) in a dose-dependent manner. This indicates that the Cf-GP induced inhibits degradation of IκB. Therefore, inhibition of the translocated NF-κB is by the stable NF-κB/IκB complex.

### Cf-GP dose-dependently downregulates the expression of integrins

**Figure 6.**
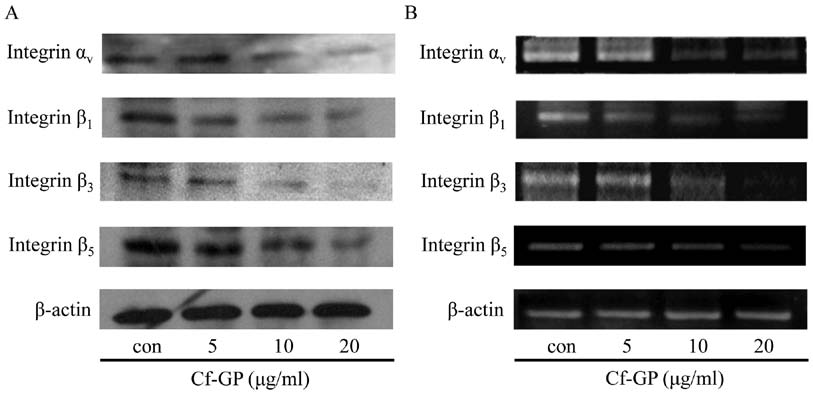
Cf-GP dose-dependently downregulates the expression of integrins α _ν_ , β _1_ , β _3_ and β _5_ . Protein and mRNA expression levels of integrins α _ν_ , β _1_ , β _3_ and β _5_ in Cf-GP-treated (5, 10 and 20 *μ* g/ml) AGS cells were determined by (A) western blot analysis and (B) RT-PCR analysis, respectively, as described in Materials and methods.

Integrin proteins play important roles in cancer cells growth and migration. Also, involvment in cancer cells angiogenesis, invasion and incessant proliferation was induced. Integrins are a family of heterodimers of α and β subunit, and cancer cells of the specific expression of each subunit have been reported ( 22 ) . Among them, integrin α _ν_ , β _1_ , β _3_ and β _5_ are known to be overexpressed in gastric cancer cells ( 23 ) . We have confirmed inhibition of growth related factor and transcription factor via reduction of TGF-β _1_ by Cf-GP. Therefore, we performed expression of integrin proteins and mRNA levels through western blot analysis and RT-PCR. The same conditions as in the treatment by Cf-GP (5, 10 or 20 *μ* g/ml) for 24 h, were used. The protein ( Fig. 6A ) and mRNA expression levels ( Fig. 6B ) of integrins α _ν_ , β _1_ , β _3_ and β _5_ were reduced by Cf-GP treatment, in a dose-dependent manner.

## Discussion

Gastric cancer is common in many countries, particularly in Asia, and is caused by irregular eating habits, stress and environmental factors. Several studies ( 24 – 27 ) have reported the anticancer effects of various marine algae, including the green alga *C. fulvescens* , which is commonly consumed in Asia. We previously observed that a glycoprotein of *C. fulvescens* (Cf-GP) induced apoptosis in AGS human gastric cancer cells through the Fas signaling pathway ( 6 , 7 ) .

In the present study, we showed that Cf-GP also inhibits the migration and proliferation of AGS cells, as well as down-regulates the expression of several growth-related proteins, including TGF-β _1_ , FAK, PI3K, AKT and the small GTPases Rho A, Rac-1 and Cdc 42. Cf-GP treatment also resulted in the downregulated expression of NF-κB, IκB, and integrins α _ν_ , β _1_ , β _3_ and β _5_ . Conversely, Cf-GP upregulated the expression of the small GTPase Rho B, which is involved in the inhibition of cell growth and the induction of apoptosis.

TGF-β _1_ is a secreted cytokine that participates in the regulation of cell migration, growth, apoptosis and differentiation. TGF-β _1_ acts through its receptor to induce FAK expression in several cancers, to activate the PI3K/AKT/small GTPase pathway ( 13 ) , and to upregulate integrin receptor expression ( 28 ) . The small GTPases Rho A, Rho B, Rac-1, and Cdc 42 are involved in signaling pathways associated with cell proliferation and migration in response to various growth factor receptors ( 13 ) . They also activate NF-κB, which induces the expression of integrin receptors, thereby contributing to cell migration. Integrin receptors are critical for cell adhesion and cell attachment-dependent growth, migration, invasion and metastasis ( 29 , 30 ) . In particular, integrin has α and β, two subunits of hetero-copolymer and these are associated with the progression of a variety of human cancer cells. Attachment in independent growth is a characteristic of transformed cells, but cancer cell growth and migration depend on the interaction of the cell adhesion receptor integrins and matrix. Therefore, integrins promoted cell proliferation by attachment-dependent effects.

Specific integrin heterodimers are expressed in different cancer cells, and the growth and migration of the cells depend on the interactions between the integrins and their extracellular ligands.

First, we observed the effects of Cf-GP in AGS cell viability by MTS assay. Treatment by Cf-GP (5, 10 or 20 *μ* g/ml) for 24 h inhibited the AGS cell growth and at the highest concentration of 20 *μ* g/ml in the apoptotic cells by approximately 50% reduction compared to control group and no toxicity to IEC-6 normal cell ( Fig. 1 ). We used a wound-healing assay to confirm cell migration and observed the denuded zone through a microscope. The gap (denuded zone) between the cells was inhibited dose-dependently by Cf-GP ( Fig. 2 ). In addition, we performed Annexin V staining assay for cell apoptosis rate by Muse Annexin V and Dead Cell kit. The apoptosis rate increased 42.68% in final Cf-GP concentration (20 *μ* g/ml) compared with the control group ( Fig. 3 ). We found TGF-β _1_ decreased FAK/PI3K/AKT/small GTPase expression by using western blot analysis and RT-PCR ( Fig. 4 ). This result shows that the inhibition of FAK inhibited TGF-β _1_ . Increased cancer cell proliferation was observed when FAK/PI3K/AKT/small GTPase were activated. Cf-GP-treated cells exhibited significant downregulation of FAK/PI3K/AKT/small GTPase proteins (Rho A, Rac-1, Cdc 42) and mRNA levels compared to the control group, but activation of Rho B induced cancer cells apoptosis. In this study, Rho B increased by Cf-GP ( Fig. 4 ). The results of the transcription factor NF-κB and IκB decreased due to inhibition of these growth factors. Activation of the NF-κB and IκB induces promotion of cancer cell migration and variety of intracellular factors ( 31 ) . We found upregulation of IκB induced downregulation NF-κB through inhibition of the growth factor and TGF-β _1_ by the Cf-GP effects ( Fig. 5 ). The integrin α _ν_ pair with multiple integrin subunit β (β _1_ , β _3_ , β _5_ ) and upregulation of integrin proteins induced migration of cancer cells. The integrin expression through the activation of transcription factors to increase was seen from previous experiment, thus, the reduction of the NF-κB and increase of IκB were indentified. Therefore, we performed expression of integrins, and confirmed that Cf-GP induced downregulation of integrin α _ν_ , β _1_ , β _3_ and β _5_ ( Fig. 6 ).

Collectively, our findings suggest that Cf-GP inhibits AGS gastric cancer cell migration and proliferation by downregulating integrin expression via the inhibition of TGF-β _1_ -activated FAK/PI3K/AKT pathways. Cf-GP may be an important factor in the development of functional foods and therapeutic agents.

## Figures and Tables

**Table I.** Oligonucleotide sequences of the primer pairs used for RT-PCR.

Name	Sequence of primers (5′→3′)
TGF-β _ 1 _	S: GCA-GAA-CCC-AAA-AGC-CAG-AGT-G
	A: CCA-TAA-CTA-CCG-TGG-AGG-TTG-A
FAK	S: TTC-ATT-ATT-TTG-AAA-GCA-ATA-GT
	A: CAA-CCC-AAC-TTC-AAA-GCA-ATT-TC
Rho A	S: CTC-ATA-GTC-TTC-AGC-AAG-GAC-CAG-TT
	A: ATC-ATT-CCG-AAG-ATC-CTT-CTT-ATT
Rho B	S: ATG-GCG-GCC-ATC-CGC-AAG-AAG-C
	A: TCA-TAG-CAC-CTT-GCA-GCA-GTT-G
Rac-1	S: GGA-CAC-AGC-TGG-ACA-AGA-AGA
	A: GGA-CAG-AGA-ACC-GCT-CGG-ATA
Cdc 42	S: CGA-CCG-CTA-AGT-TAT-CCA-CAG
	A: GCA-GCT-AGG-ATA-GCC-TCA-TCA
PI3K	S: AGG-AGC-GGT-ACA-GCA-AAG-AA
	A: GCC-GAA-CAC-CTT-TTT-GAG-TC
Akt	S: CAA-CTT-CTC-TGT-GGC-GCA-GTG
	A: GAC-AGG-TGG-AAG-AAC-AGC-TCG
IκB	S: TGG-ATG-AAC-TGC-GTG-GTG-CAG
	A: GCA-GAA-GTG-TCC-CTG-TTC-CAG
NF-κB	S: TCA-GGG-AAT-ATC-CAC-CTA-TCA-CTT-CAG
	A: CAT-CAG-CAG-CAG-CCA-TGT-ACT-CTT-CAC
Integrin α _ ν _	S: GAA-GCT-TCA-TCT-CCA-GTC-CCT
	A: TGG-GTA-GGG-CTG-TTT-GTC-ATC-ATA
Integrin β _ 1 _	S: GAC-CTG-CCT-TGG-TGT-CTG-TGC
	A: AGC-AAC-CAC-ACC-AGC-TAC-AAT
Integrin β _ 3 _	S: CCC-TCG-AAA-ACC-CCT-GCT-AT
	A: TTA-GCG-TCA-GCA-CGT-GTT-TGT-AG
Integrin β _ 5 _	S: GGC-TGG-GAC-GTC-ATT-CAG-AT
	A: AGC-TGG-AAG-GTG-GTC-TTG-TCA
β-actin	S: CGT-ACC-ACT-GGC-ATC-GTG
	A: GTG-TTG-GCG-TAC-AGG-TCT-TTG

S, sense; A, antisense.
